# ﻿Sexual dimorphism of feeding stylets in some Thysanoptera – Phlaeothripinae, with description of two new species of *Haplothrips* from China

**DOI:** 10.3897/zookeys.1176.105751

**Published:** 2023-08-22

**Authors:** Lihong Dang, Laurence Mound

**Affiliations:** 1 School of Bioscience and Engineering, Shaanxi University of Technology, Hanzhong, 723000, China Shaanxi University of Technology Hanzhong China; 2 Shaanxi Province Key Laboratory of Bioresources, Hanzhong, 723000, China Shaanxi Province Key Laboratory of Bioresources Hanzhong China; 3 Qinba Mountain Area Collaborative Innovation Center of Bioresources Comprehensive Development, Hanzhong, 723000, China Qinba Mountain Area Collaborative Innovation Center of Bioresources Comprehensive Development Hanzhong China; 4 Qinba State Key Laboratory of Biological Resources and Ecological Environment (Incubation), Hanzhong, 723000, China Qinba State Key Laboratory of Biological Resources and Ecological Environment (Incubation) Hanzhong China; 5 Australian National Insect Collection CSIRO, PO Box 1700, Canberra, ACT 2601, Australia Australian National Insect Collection CSIRO Canberra Australia

**Keywords:** Haplothripini, *Haplothripshelanshanensis*, *H.longistylus*, maxillary stylets

## Abstract

Sexual dimorphism in feeding stylets is recorded among some Phlaeothripinae that have maxillary stylets long and close together in females but wider apart in males. These atypical long feeding stylets have been found in two new species of *Haplothrips* from China, both taken on *Artemisia* from Plateau zone. Two species are described and illustrated: *H.helanshanensis***sp. nov.** from Helanshan Mountain and *H.longistylus***sp. nov.** from Tibet. There is no evidence of any association between feeding behaviors and feeding stylet orientation.

## ﻿Introduction

Sexual dimorphism is widespread throughout the insect order Thysanoptera, such that it is likely to have been a plesiotypic condition inherited from the ancestors of the group. It is recorded in species from Cretaceous amber from Burma (Ulitzka 2022) and occurs in both suborders of living thrips, including members of families that are generally considered to be the least derived, such as Merothripidae and Aeolothripidae. Sexual dimorphism can be so marked that Trybom (1912) erected *Mitothrips* for two males of a species that was subsequently recognized as a species of *Franklinothrips* (Back 1912). In this genus, females are ant-like in appearance and behavior, whereas males are slender, as in most Aeolothripidae. Sexual dimorphism is generally associated with some difference in the behavior between the sexes, particularly in mating behavior (Mound 2005). In contrast, the purpose of this article is to discuss a much less obvious example of sexual dimorphism involving differences between the sexes in the position of the feeding stylets.

### ﻿Sexual dimorphism in feeding stylet positions

**Figures 1–8.**
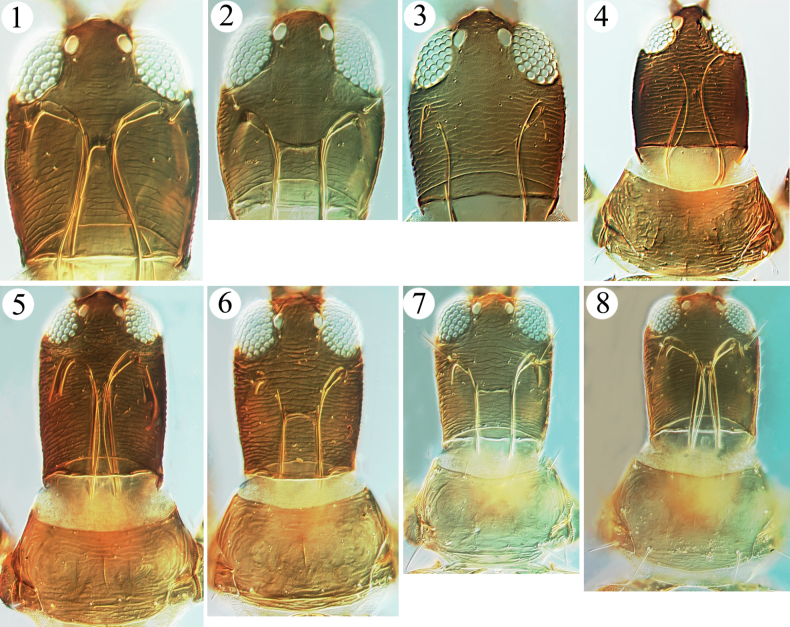
*Haplothrips* spp. Heads of *H.stofbergi* (**1, 2**) **1** female **2** male; *H.timori* (**3, 4**) **3** head, male **4** head and pronotum, female; head and pronotum of *H.helanshanensis* sp. nov. (**5, 6**) **5** female **6** male; head and pronotum of *H.longistylus* sp. nov. (**7, 8**) **7** male **8** female.

This curious example of sexual dimorphism came to the attention of the authors in describing below two new species of *Haplothrips* from China. In these species the feeding stylets of females are close together medially on the head, whereas those of males are positioned considerably further apart (Figs 1–8). At present there are no observations to help suggest why these males and females should have their feeding stylets positioned differently on the head, although an obvious correlation might be the precise feeding site, such as in flowers versus on leaves.

Bhatti (1979) was the first person to record a thrips species as having the maxillary stylets of females closer together than those of conspecific males. He recorded that the separation between the maxillary stylets of females ranged from 13–19 μm in contrast to 30–40 μm in males in *Ananthakrishnanaeuphorbiae* (Priesner). Subsequently, Bhatti (1997) indicated a similar situation in another species of Haplothripini that he described from India. In this species, *Apterygothripsbanyan* Bhatti, the distance between the maxillary stylets of females he gave as 30–38 μm, in contrast to 37–39 μm in males. The only other published reference to this type of sexual dimorphism among thrips is by Okajima and Urushihara (1995). These authors illustrated from Thailand the previously unknown male of *Stephanothripsoccidentalis* Hood & Williams, a species that is distantly related within Phlaeothripidae to the two Haplothripini discussed above. Females of *S.occidentalis* have been found worldwide and have the stylet separation about 25 μm, but the males from Thailand have the stylets 34–39 μm apart.

Whilst studying the two new *Haplothrips* species described below, further instances of maxillary stylet position varying between sexes were observed in two species of this genus available in the Australian National Insect Collection. The first, *Haplothripsstofbergi* Faure, was described from South Africa on various grasses (Faure 1958), but the lengthy description did not include any comparison of stylet position between the sexes. However, 10 females and 10 males of this species have been studied in ANIC that were taken in quarantine in Australia from grasses collected in South Africa. The species can be distinguished from other *Haplothrips* species by having only three sense cones on the fourth antennal segment. Females of these specimens have the maxillary stylets about 25 μm apart, but males have this separation about 40 μm (Figs 1, 2). The second species, Haplothrips (Trybomiella) timori Mound & Minaei was described from Darwin in northern Australia based on a single male (Mound and Minaei 2007). Subsequently, one female and three males were collected on Badu Island in the Torres Strait, two females were taken on Timor Leste at Dili, and two females with one male have been seen from near Kuala Lumpur, Malaysia. The species is possibly associated with the widespread weed, *Euphorbiahirta*. In females the maxillary stylet separation is about 25 μm, but in males it is nearer 50 μm (Figs 3, 4).

## ﻿Material and method

For study, thrips specimens are mounted onto microscope slides usually in Canada balsam (ThripsWiki 2023). The body contents of each specimen are generally cleared using a weak solution of sodium hydroxide in order that surface sculpture can be seen clearly. This process requires considerable patience because the stylets become disrupted out of their natural position if the hydroxide solution is too strong or if the specimens are heated. The descriptions and photomicrograph images are produced from slide-mounted specimens with a Leica DM2500 using DIC illumination and processed with Automontage and Photoshop software. The abbreviations used for the pronotal setae are as follows: am – anteromarginal, aa – anteroangular, ml – midlateral, epim – epimeral, pa – posteroangular. The unit of measurements in this study is micrometre. All specimens studied here are deposited in the School of Bioscience and Engineering, Shaanxi University of Technology (**SNUT**), Hanzhong, China, and in Australian National Insect Collection (**ANIC, CSIRO**), Canberra, Australia.

## ﻿Taxonomy

### ﻿ Haplothrips helanshanensis

Taxon classificationAnimaliaThysanopteraPhlaeothripidae

Dang & Mound sp. nov.

8F80F908-51E2-5E15-AA1B-B6EB584D2704

https://zoobank.org/ED751D73-E3C4-477B-8324-9086CD996E81

Figs 5 , 6 , 9 , 11 , 13 , 15 , 17 , 18

#### Materials examined.

***Holotype***, ♀ (SNUT), China, Inner Mongolia, Helan Mountain National Nature Reserve, on *Artemisia* sp., 04.viii.2010, L.H. Dang. ***Paratypes***, 2♀2♂ (SNUT), with the same data as holotype; 1♀1♂ (ANIC), with the same data as holotype.

#### Description.

**Figures 9–20.**
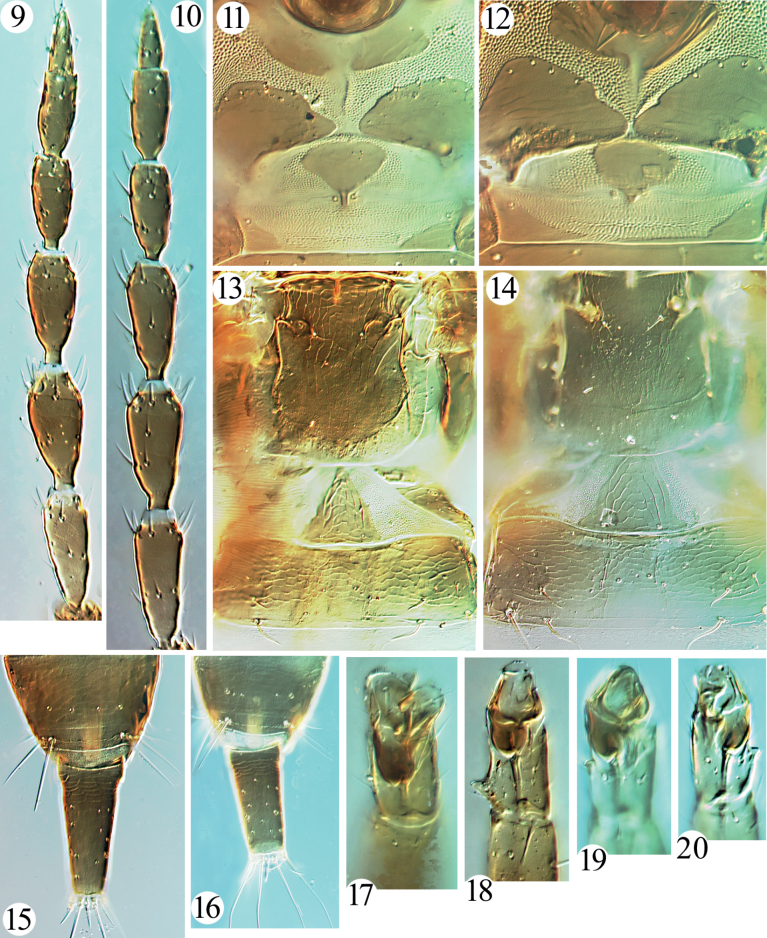
*Haplothrips* spp. antennae (**9, 10**) **9***H.helanshanensis* sp. nov. **10***H.longistylus* sp. nov.; prosternum and mesopresternum (**11, 12**) **11***H.helanshanensis* sp. nov. **12***H.longistylus* sp. nov.; metanotum and tergites I–II (**13, 14**) **13***H.helanshanensis* sp. nov. **14***H.longistylus* sp. nov.; tergites IX–X (**15, 16**) **15***H.helanshanensis* sp. nov. **16***H.longistylus* sp. nov.; fore tarsal tooth of *H.helanshanensis* sp. nov. (**17, 18**) **17** female **18** male; fore tarsal tooth of *H.longistylus* sp. nov. (**19, 20**) **19** female **20** male.

**Female macroptera.** Body brown. All legs brown, with brownish yellow on fore tarsi and extreme apices of fore tibiae. Antennal segments uniformly brown, with III yellowish brown (Fig. 9). Wings and body setae hyaline.

***Head*.** Head elongate, about 1.5 times as long as wide (Fig. 5); dorsal surface sculptured with transverse striae; eyes small, about one-quarter of head length, postocular setae minute, similar to other cheek setae (Fig. 5); cheeks parallel. Mouth-cone rounded, maxillary stylets long, retracted into postocular setae, close together, the narrowest separation about 5 μm, bridge stout and short, 15 μm wide (Fig. 5). Antennae 8-segmented, sense cones on segments III–IV small, about half breadth of this segment, III with 1+1, IV with 2+2 (Fig. 9).

***Thorax*.** Pronotum weakly sculptured, notopleural sutures complete (Fig. 5); am, aa and ml minute, epim and pa setae developed, pointed at apex, epim the longest (Fig. 5); mesopresternum eroded medially, reduced to two small, lateral, triangular plates (Fig. 11); metanotum weakly reticulate at middle and smooth laterally (Fig. 13), metathoracic sternopleural sutures absent. Fore tarsi with a tiny tooth (Fig. 17).

***Abdomen*.** Pelta acutely triangular and weakly reticulate, with a pair of campaniform sensilla (Fig. 13); abdominal tergites II–VII with two pairs of wing-retaining setae; S1–S3 on tergite IX short, much shorter than tube, pointed at apex (Fig. 15); tube about 0.4 times as long as head, 1.8 times as long as basal width, anal setae shorter than tube.

***Measurements*** (holotype female in μm). Body length 2050. Head length 255, width just behind eyes 175; eye length 60, postocular setae length 10; the narrowest separation between maxillary stylets 5, width of bridge 15. Antenna length 370, segments I–VIII length (widest) 35(30), 50(32), 60(32), 60(30), 60(30), 45(25), 40(20) and 30(12), sensoria on segment III length 15. Fore wing length 88. Pronotum length125, width 235, length of pronotal setae, am 5, aa 5, ml 5, epim 23, pa 15. Pelta length 95, width 85; tergite IX posteromarginal setae S1–S3, 75, 65, 45; tube length 105, basal width 60, apical width 35; anal setae length 85.

**Male macroptera.** Similar to female; but maxillary stylets wider apart, about one-quarter of head width (Fig. 6); postocular setae slightly longer than other cheek setae; fore tarsal tooth bigger than in females (Fig. 18); abdominal tergite IX setae S2 short and stout, sternites without a pore plate.

***Measurements*** (paratype male in μm). Body length 1820. Head length 225, width just behind eyes 160; eye length 70, postocular setae length 15; the narrowest separation between maxillary stylets 40, width of bridge 40. Antenna length 375, segments I–VIII length (widest) 30(30), 35(25), 55(22), 55(30), 55(25), 45(20), 45(20) and 30(12), sensoria on segment III length 15. Pronotum length 115, width 220, length of pronotal setae, am 10, aa 10, ml 10, epim 25, pa 20. Fore wing 80. Pelta length 80, width 75; tergite IX posteromarginal setae S1–S3, 85, 35, 80; tube length 130, basal width 55, apical width 35; anal setae length 85.

#### Etymology.

This species name is based on its collecting location.

#### Comments.

This new species is similar to the Australian species, *H.salicorniae* Bournier, in having the postocular setae minute, mesopresternum eroded medially, antennal segments III–IV with two and four sense cones respectively, S1 on tergite IX much shorter than tube, fore tarsal tooth tiny in female, metanotum sculptured with reticulation, and the colour pattern of legs and antennae. In contrast, because the fore wings of *H.salicorniae* have no duplicated cilia, that species is placed in the subgenus Trybomiella. The new species from China has several duplicated cilia on fore wings and is placed in the subgenus Haplothrips. In addition, in *H.salicorniae* the pronotum has three pairs of well-developed setae (aa, epim and pa) that are expanded at the apex, whereas in *H.helanshanensis* sp. nov. these setae are pointed at the apex and the aa and pa are minute (Figs 5, 6). In both species the females have similarly shaped heads and maxillary stylets, but unfortunately, males of *H.salicorniae* remain unknown (Mound and Minaei 2007).

### ﻿ Haplothrips longistylus

Taxon classificationAnimaliaThysanopteraPhlaeothripidae

Dang & Mound sp. nov.

3F9870C7-56BE-51DC-85FB-073AF3AE9352

https://zoobank.org/983CCC68-5539-4486-BC33-DAB3AE3AEE88

Figs 7 , 8 , 10 , 12 , 14 , 16 , 19 , 20

#### Materials examined.

***Holotype***, ♀ (SNUT), China, Tibet, Lasa city, Nanshan Park, on *Artemisiagmelinii*, 03.viii.2019, L.H. Dang. ***Paratypes***, 1♀1♂ (SNUT), with the same data as holotype; 1♀1♂ (ANIC), with the same data as holotype.

#### Description.

**Female macroptera.** Body brown. All legs brown with fore tarsi and extreme apices of fore tibiae brownish yellow. Antennal segments uniformly brown, III brown with pale at base (Fig. 10). Wings hyaline with brown around subbasal setae, body setae hyaline.

***Head*.** Head elongate, about 1.3 times as long as wide (Fig. 8); dorsal surface weakly sculptured with transverse striae; eyes small, about one-quarter of head length, postocular setae well developed, about as long as eyes (Fig. 8); cheeks almost parallel. Mouth-cone rounded, maxillary stylets long, retracted to postocular setae, close together, the narrowest separation 5 μm, bridge stout and short, 15 μm wide apart (Fig. 8). Antennal 8-segmented, sense cones on segments III–IV small, about half as broad as its segment, III with 1+1, VI with 2+2 (Fig. 10).

***Thorax*.** Pronotum almost smooth, notopleural sutures complete (Fig. 8); five pairs of major setae well developed, pointed at apex, pa the longest (Fig. 8); mesopresternum strongly eroded medially, reduced to two small, lateral, triangular plates (Fig. 12); metanotum very weakly reticulate at middle and smooth laterally, metathoracic sternopleural sutures absent. Fore tarsi with a tiny tooth (Fig. 19).

***Abdomen*.** Pelta triangular and weakly reticulate, with a pair of campaniform sensilla (Fig. 14); abdominal tergites II–VII with two pairs of wing-retaining setae; S1–S3 on tergite IX short, much shorter than tube, pointed at apex (Fig. 16); tube about 0.6 times as long as head, 2.0 times as long as basal width, anal setae shorter than tube.

***Measurements*** (holotype female in μm). Body length 2450. Head length 215, width just behind eyes 160; eye length 65, postocular setae length 65; the narrowest separation between maxillary stylets 10, width of bridge 15. Antenna length 410, segments I–VIII length (widest) 35(35), 50(30), 62(30), 60(30), 60(30), 50(25), 45(20) and 30(12), sensoria on segment III length 15. Fore wing length 1070. Pronotum length160, width 280, length of pronotal setae, am 35, aa 35, ml 30, epim 55, pa 60. Pelta length 100, basal width 130; tergite IX posteromarginal setae S1–S3, 75, 75, 80; tube length 135, basal width 65, apical width 40; anal setae length 100.

**Male macroptera.** Similar to female; but smaller, maxillary stylets wide apart, about one-third of head width (Fig. 7); fore tarsal tooth small (Fig. 20); abdominal tergite IX setae S2 short and stout, sternites without a pore plate. One larger male with body length 2050, its separation between maxillary stylets 25 μm, about one-seventh of head width.

***Measurements*** (paratype male in μm). Body length 1720. Head length 200, width just behind eyes 155; eye length 65, postocular setae length 55; the narrowest separation between maxillary stylets 50, width of bridge 50. Antenna length 360, segments I–VIII length (widest) 35(30), 40(25), 55(22), 55(25), 55(25), 45(20), 45(20) and 30(12), sensoria on segment III length 15. Pronotum length 120, width 200, length of pronotal setae, am 25, aa 30, ml 30, epim 50, pa 50. Fore wing length 760. Pelta length 75, width 80; tergite IX posteromarginal setae S1–S3, 75, 35, 95; tube length 125, basal width 55, apical width 45; anal setae length 95.

#### Etymology.

This species name refers to the elongate maxillary stylets.

#### Comments.

The new species is similar to *H.pharao* Priesner from Egypt in having major setae pointed at apices, postocular setae slightly shorter than eyes, the mesopresternum divided into two lateral triangles, and the fore tarsal tooth tiny in females. However, it can be differentiated in colour pattern of antennae and legs and length of S1 on tergite IX. In *H.longistylus* sp. nov., all legs are brown except fore tibiae with extreme apices and fore tarsi brownish yellow, antennal segments are uniformly brown, but III pale at base (Fig. 10) and S1 on tergite IX is much shorter than tube (Fig. 16) (in *H.pharao*, at least all tarsi yellow, and antennal segments III–IV uniformly yellow, IV–VI yellow with brown at apex, and S1 on tergite IX about as long as tube). The new species is also similar to *H.stofbergi* from Africa in the shape of the major setae, mesopresternum, fore tarsal tooth, S1 on tergite IX, and pelta, but it differs in having metathoracic sternopleural sutures absent and antennal segments III–IV with two and four sense cones, respectively (Fig. 10), whereas in *H.stofbergi* the metathoracic sternopleural sutures are very long and antennal segments III–IV have one and three sense cones. Only one female of *H.pharao* is available in ANIC, and maxillary stylets of the male were not mentioned in the original description (Priesner 1930), but both sexes of *H.stofbergi* are checked here, and sexual dimorphism in the maxillary stylets confirmed (Figs 1, 2).

## Supplementary Material

XML Treatment for Haplothrips helanshanensis

XML Treatment for Haplothrips longistylus

## ﻿Additional information

### Author ORCIDs

Lihong Dang https://orcid.org/0000-0002-7571-8426

Laurence Mound https://orcid.org/0000-0002-6019-4762

### Data availability

All of the data that support the findings of this study are available in the main text.
